# The Sanatorium Treatment of Pulmonary Tuberculosis—Is It a Success?

**Published:** 1907-03

**Authors:** D. J. Chowry Muthu

**Affiliations:** Physician, Mendip Hills Sanatorium, Wells.


### THE SANATORIUM TREATMENT OF PULMONARY TUBERCULOSIS—IS IT A SUCCESS?

BY

D. J. CHOWRY MUTHU, M.D.,

*Physician, Mendip Hills Sanatorium, Wells.*

SUCH a mass of literature has been written on pulmonary tuberculosis and its treatment, that I will not trouble the reader by going over any unnecessary ground, but shall confine my remarks to personal observations made during my eight years' connection with the sanatorium treatment of consumption. The pioneers of the open-air movement, most of whom are medical men belonging to various sanatoria, have cause to be proud of the success of their efforts in creating in this country a public interest in the subject. The man in the street, encouraged by the early enthusiasts of the movement, expected that everyone entering the portals of a sanatorium would be cured in a very short time. And even medical men, without giving a due consideration to the question, were led away by exaggerated reports, and proclaimed in private and in public that a few weeks' stay in an open-air establishment would be sufficient to cure a consumptive patient. The inevitable consequence was that the results fell short of the public expectation, and discredit most unjustly fell on the whole movement.

But what are the facts ? Speaking from my own experience, I should say, to put it modestly, that in at least 50 per cent. of cases life and health are restored by the open-air treatment. That is, about half the patients of all stages that enter a sanatorium either get well completely or their disease is arrested so as to enable them to return to work.

To give a mere bald statement of figures, we have records of one hundred and fifty patients treated in my sanatorium during the first four years ending August, 1903. Of these, seventy-eight, or about 51 per cent., have been restored to health, and are following their various occupations to the present day. Surely this is a record that any treatment can be proud of, and is worth striving after.

The duration of the treatment has a very important bearing on its success. It is utterly absurd to expect, as some have done, to cure a patient or even arrest the disease in three months. There is no doubt some truth in the insinuation that the patients who have been partly patched up by the open-air treatment and return to unhealthy surroundings relapse more quickly than those who have remained in the old conditions of life, simply because half a cure is worse than no cure. A creaking door lasts longer if left alone, because everyone knows its deficiency and uses it carefully ; but it looks strong when it is half mended and painted, and the first careless knock breaks it to pieces. The open-air cure is, I am sorry to say, long and tedious, requiring infinite patience and perseverance on the part of the physician and the patient. It is Nature's cure, and Nature builds well, but slowly. The causes of failure of sanatorium treatment are mainly due to :—

(*a*) Medical men not sending their cases at an early stage.

(*b*) Patients who at the first sign of return of health go home thinking they are cured.

(*c*) Institutions which cannot keep patients for more than two to four months, and then send them back to their former unhygienic surroundings.

(*d*) Treating patients at home when no medical supervision or discipline is possible.

The most important cause of failure in my experience is that patients either have no means to continue the treatment for long, or have not the patience to persevere in the sanatorium *regime*. At the first sign of return of health they leave the sanatorium and seek more congenial surroundings. Rigid discipline, cold, absence from home and friends, &c., are unpalatable, and it is most difficult to convince patients that health can only be got back by great sacrifice, that Nature demands strict conformity to her laws. It is one of the signs of this decadent age that patients have not the strength of character or stamina to stand fast and strenuously continue in the fight with the disease.

I go further, and say that the question of arrest of the disease is more a matter of a patient's perseverance in the treatment than even whether he comes under treatment at the first, second or any stage of the disease. This may be a bold statement to make, but nevertheless it is my experience. Over and over again I have known patients who came to the sanatorium in the early stage, and who returned home too soon, partly patched up, with the result that after a few months they succumbed to the disease. On the other hand, I have known others who came to be treated in the cavity stage and with very little resisting power, but who have persevered for two and three winters, and are living now to testify to the efficacy of the open-air treatment. Of course I exclude from the present consideration those cases which seem to go wrong from the very beginning, and which get steadily worse in spite of every care and attention. We do not understand at present the laws that underlie these cases ; some day we shall. I also exclude those cases of advanced stage, where gastric functions are deranged, and the process of assimilation and excretion is poisoned by toxins and other products of pathogenic organisms. But apart from these cases, broadly speaking, the failure or success of the treatment depends more or less upon the patient himself. In other words, the amount of vitality and fighting force determine the prognosis of almost every case. The sanatorium treatment increases the resisting force of the patient. If he perseveres in the treatment, there is every probability of his increasing his vital force and of overcoming the disease sooner or later.

On the other hand, I foresee that the sanatorium treatment will fail, because patients, from lack of time or means, from lack of inclination or strenuousness, will desire to get well in a hurry, and cannot. There is no short cut to Nature's cure; she works slowly, but surely. The earth takes just as long to go round the sun as it did some hundreds of years ago, and it has found no short road yet. Various remedies have been brought forward from time to time and exploited as speedy cures for the disease, but they all have been failures so far. Nature cannot be tricked to cure a patient in this way. She proceeds step by step, line upon line, and health can only be regained by strictly adhering to her laws; and the result of perseverance is a sure and certain success in many cases.

The success of the open-air movement is not merely to be gauged by the number of lives it has been the means of saving, but in a greater degree it is seen by the way in which it opens up larger issues, and rouses the nation to see the evil tendencies of modern thought and modern life, suggesting reforms along national, social and municipal lines. Tuberculosis is one of the evils of present-day civilisation. As long as there are crowded centres like London, Manchester and Liverpool, which are breeding-places for dirt and microbes, one can never hope to eradicate consumption. It is like trying to bale out water from a ship that has sprung a leak. As fast, or faster than you can pump, the water rushes in through the hole and fills the ship. As fast as you can cure patients, their places are filled up by others manufactured by the crowded and dirty towns.

Besides, tuberculosis goes hand in hand with other evil factors in the vicious circle, viz. competition, overcrowding, poverty, drink, a life of bustle and excitement, all of which tend to keep man at high tension and irritation, giving him no time for the recuperation of vital forces, which are necessary for the maintenance of his health. Nature not only needs time to build, but also rest and quiet to do her work. In silence she manufactures in her secret laboratories the vital energies required to maintain life's functions and duties. Mere giving attention to sanitation, or building large sanatoria for consumption, is like sprinkling carbolic powder over the leakage of drain pipes. The sanatorium treatment is only a part of a great movement which aims at going to the root of the matter. Like the ever-widening circles caused by a stone dropped into a still lake, it tends to widen the outlook and extend its reforms from one disease to the prevention of all disease, and never rests till it has taught the nation to regard the health of its citizens as a sacred heritage, which it should guard at all costs and against all enemies. Looking thus from a broad point of view, the sanatorium treatment can never be regarded as a failure.

